# Exploring the Role of Plant-Based Nutrition in Polycystic Kidney Disease

**DOI:** 10.3390/nu17223518

**Published:** 2025-11-11

**Authors:** Ahmad Sarmad, Niloufar Ebrahimi, Fouad T. Chebib, Pranav S. Garimella, Diana Bruen, Amir Abdipour, Sayna Norouzi

**Affiliations:** 1 Division of Nephrology, Rush University Medical Center, Chicago, IL 60612, USA; 2 Department of Medicine, Division of Nephrology, Loma Linda University Medical Center, Loma Linda, CA 92354, USA; 3 Division of Nephrology and Hypertension, Mayo Clinic, Jacksonville, FL 32224, USA; 4 Division of Nephrology and Hypertension, Department of Medicine, University of California San Diego, San Diego, CA 92093, USA; 5 Renal Dietitian/Specialist in PKD, The PKD Dietitian, Henrico, VA, USA

**Keywords:** autosomal dominant polycystic kidney disease, ADPKD, polycystic kidney disease, PKD, plant-based diet

## Abstract

Polycystic kidney disease (PKD) is a genetic disorder characterized by progressive kidney cyst formation and eventual kidney failure. Emerging evidence suggests that plant-based diets may confer therapeutic benefits in patients with PKD by modulating mTOR and AMPK signaling, reducing oxidative stress and inflammation, while improving metabolic and cardiovascular profiles. These diets, which are low in animal protein and rich in antioxidants, may help lower uric acid levels and support blood pressure control, thereby potentially reducing fibrosis. However, careful planning is required to avoid nutritional deficiencies. Well-designed clinical trials are warranted to validate their role in ADPKD management.

## 1. Introduction

### 1.1. Definition and Prevalence

Polycystic Kidney Disease (PKD) is a genetic disorder characterized by the formation of numerous fluid-filled cysts in the kidneys, leading to progressive kidney enlargement and failure over time. According to its inheritance pattern, PKD is classified into two main types: Autosomal Dominant PKD (ADPKD) and Autosomal Recessive PKD (ARPKD) [1].

ADPKD is the more common form, affecting approximately 1 in 1000 people globally. It typically manifests later in life during adulthood, with cysts gradually increasing in size and number, potentially causing high blood pressure, kidney infections, and, eventually, kidney failure [1,2].

Cyst development in PKD disrupts normal kidney architecture and impairs nephron function, contributing to complications such as hypertension, hematuria, and nephrolithiasis. As the disease progresses, kidney function declines, often resulting in kidney failure, which may require dialysis or kidney transplantation for survival [3].

### 1.2. Pathophysiology

Mutations in *PKD1* (located on chromosome 16) account for about 78% of ADPKD cases, and this form is often more severe, with earlier onset of symptoms and faster progression to kidney failure. *PKD2* mutations (located on chromosome 4) are responsible for the remaining 15% of cases and are generally associated with a more gradual disease course [3,4]. However, the genetic landscape of ADPKD has broadened considerably. Beyond these two major genes, at least six minor genes, including *IFT140*, *ALG5*, *ALG9*, *NEK8*, *DNAJB11*, and *GANAB*, have been implicated in cystic kidney disease. Moreover, a growing number of additional mutations, including those affecting developmental pathways, type IV collagen genes, autosomal dominant tubulointerstitial kidney disease genes, nephronophthisis genes, syndromic or tumor-related genes, and autosomal dominant polycystic liver disease genes, can present with a phenotype that includes bilaterally enlarged cystic kidneys [5].

Polycystin proteins play a central role in ADPKD. Polycystin-1 is involved in cell–cell and cell–matrix interactions, while polycystin-2 functions as a calcium channel [3,6]. They are found in the primary cilium, with polycystin-1 localized in the plasma membrane or endoplasmic reticulum and polycystin-2 primarily localized in the endoplasmic reticulum [7]. Together, these proteins regulate intracellular calcium signaling, which is critical for maintaining the structure of kidney tubular cells and homeostasis [8]. When these proteins are defective, the normal regulation of cell proliferation and fluid secretion is disrupted, leading to abnormal cyst formation [6,8]. The hallmark of ADPKD is the progressive formation of cysts in the kidneys, which begins during early embryonic development. Symptoms, however, often remain undetected until adulthood [1]. The cysts arise from different segments of the nephron, and their expansion over time leads to kidney enlargement and progressive loss of kidney function [3,6]. The disrupted calcium signaling also causes increased cyclic AMP (cAMP) levels, which promote cyst growth and fluid secretion within the cysts [9]. Elevated cAMP levels in PKD have been linked to dysregulation of downstream signaling pathways, including activation of protein kinase A (PKA) and cyclins, which promote aberrant cell proliferation and fluid secretion [9]. Specifically, increased cAMP levels enhance PKA activity, which in turn activates cyclin and CDC25 while downregulating p21 [10,11]. Consequently, this cascade increases levels of certain cytokines and growth factors that contribute to cyst formation [9].

### 1.3. Importance of Dietary Management in Chronic Kidney Disease

Lifestyle and dietary interventions have long been pivotal management tools implemented by nephrologists in the setting of chronic kidney disease (CKD). According to a study, it is hypothesized that diets high in protein and animal products can place a burden on the kidneys. A high dietary acid load and animal-predominant protein intake may increase the workload on the kidneys. In patients with certain forms of CKD, acidosis emerges as a progressively worsening complication [12]. With animal-based and ketogenic diets, ketoacidosis is usually observed in patients with diabetes [13]. Nutritional ketosis resulting from carbohydrate restriction does not typically produce the markedly elevated ketone levels seen in ketoacidosis [12,14]. An elevated risk is also noted in patients taking sodium–glucose cotransporter-2 (SGLT2) inhibitors [15]. This risk is particularly pronounced with meat-based ketogenic therapy, since such diets have a high potential renal acid load (PRAL), exacerbating acid retention [16,17]. These microcrystals in kidney tubules can worsen cyst formation in patients with PKD. Furthermore, they increase the risk of kidney stone formation by creating an environment conducive to lithogenesis, characterized by low urinary pH, hypocitraturia, and enhanced microcrystal aggregation. This is particularly crucial to avoid in PKD, given the already elevated baseline risk of nephrolithiasis in this population [14,18]. In addition to dietary acid load, alcohol consumption may also influence disease progression in PKD. Although moderate intake has been variably studied in the general population, excessive alcohol intake can promote oxidative stress, worsen hypertension, and impair liver function, factors that are particularly relevant in both ADPKD and ARPKD. Furthermore, alcohol can interfere with medication adherence and nutrient absorption, potentially compromising kidney health. Thus, minimizing or avoiding alcohol intake may support overall disease management in PKD [19]. Importantly, plant-based diets may confer cardiovascular protection given the elevated cardiovascular risk among individuals with PKD [20].

## 2. Overview of Plant-Based Diets

Plant-based/vegetarian diets are those in which one’s diet consists primarily of plant-derived sources of nutrition and food rather than animal-based products [21]. There are various sub-categories of plant-based diets [22]:

- Vegan: excludes all animal products.
- Lacto-Ovo Vegetarian: includes dairy and eggs.
- Pescatarian: includes fish, seafood, dairy, and eggs.

The KDIGO 2025 ADPKD Guideline recommends a dietary protein intake of 0.8–1.0 g/kg/day for patients with ADPKD and CKD stages 1–4, emphasizing the preference for plant-based proteins over those from red and processed meats [23]. In alignment with these guidelines, the Plant-Dominant Low-Protein Diet (PLADO) model suggests a protein intake of 0.6–0.8 g/kg/day, with at least 50% of the protein derived from plant-based sources, aiming to balance nutritional needs while potentially mitigating disease progression. Recent clinical literature supports this approach to reduce CKD progression while maintaining nutritional adequacy [24].

### 2.1. Benefits Within the Nutritional Context of Plant-Based Diets

A plant-based diet offers a variety of nutritional benefits and potential risks, which vary depending on the structure of the diet. Plant-based diets are rich in essential nutrients, including fiber, vitamins (C, E, and folate), minerals (magnesium and potassium), and phytonutrients such as polyphenols and carotenoids [25]. These components are vital for supporting cellular health, immune function, and reducing oxidative stress. Fiber intake is significantly higher in plant-based diets, leading to improved digestion, better glycemic control, and a reduced risk of heart disease and colorectal cancer [26]. The abundance of antioxidants in plant foods, particularly from fruits, vegetables, and whole grains, helps to reduce inflammation and oxidative stress, contributing to lower risks of chronic diseases [27].

Plant-based diets may also improve cardiovascular health, particularly in individuals with kidney disease [28]. Plant-based diets are associated with lower incidences of hypertension and reduced risk of heart disease [29,30]. These benefits are partly attributable to their higher content of potassium and magnesium, which contribute to improved blood pressure regulation, vascular health, and cardiovascular outcomes. Such mineral-rich dietary patterns complement the broader cardiometabolic advantages of plant-based consumption [31]. The beneficial effects are mainly attributed to higher intakes of fiber, antioxidants, and unsaturated fats, alongside reduced consumption of saturated fats and cholesterol [28]. Plant-based diets can also reduce the risk of type 2 diabetes due to high fiber content, which helps regulate blood sugar levels [27,28]. Whole grains, legumes, and vegetables have a low glycemic index, indicating their ability to help avoid rapid increases in blood glucose levels. Several studies have demonstrated that a plant-based diet improves insulin sensitivity and may contribute to preventing and managing diabetes [27,28].

Moreover, it has been shown that consuming more fruits, vegetables, and legumes, which are rich in fiber and phytochemicals, is associated with a reduced risk of certain types of cancer, particularly colorectal, breast, and prostate cancer [32]. The protective effects are thought to result from the combination of antioxidants, anti-inflammatory compounds, and fibers that modulate gut microbiota and overall immune response [32]. Individuals on plant-based diets tend to have lower body mass index (BMI) and less body fat compared to those on omnivorous diets. The high fiber content, nutrient density, and lower energy density of plant-based foods contribute to a greater sense of fullness, which helps in reducing overall calorie intake [33].

### 2.2. Risks Within Nutritional Context of Plant-Based Diets

Vitamin B12, crucial for red blood cell formation and neurological function, is found almost exclusively in animal products. Individuals adhering to a strict plant-based diet, especially vegans, are at risk of B12 deficiency, which can lead to anemia and neurological issues [34]. Supplementation or the consumption of fortified foods is recommended to mitigate this risk [34]. Furthermore, while plant-based foods such as lentils, beans, and spinach contain iron, the non-heme iron found in plants is less bioavailable than the heme iron from animal sources [35]. This can increase the risk of iron deficiency, particularly in individuals with higher iron needs, such as menstruating women and athletes [35]. Including vitamin C-rich foods alongside plant sources of iron can improve absorption [35].

Plant-based diets often provide insufficient amounts of long-chain omega-3 fatty acids—eicosapentaenoic acid (EPA) and docosahexaenoic acid (DHA)—which are primarily found in fish [36]. These fatty acids are essential for brain health and reducing inflammation [36]. While ALA (alpha-linolenic acid), the plant-based omega-3 precursor found in flaxseeds and chia seeds, can convert to EPA and DHA, the conversion rate is inefficient [36]. Supplementing with algae-based omega-3 can help meet the requirements. In individuals who follow vegan or vegetarian diets for ethical or religious reasons, algae-based omega-3 is the preferred option. For others, fish-based omega-3 supplements may be recommended, as they provide the active forms EPA and DHA more directly and efficiently than plant-derived precursors [37]. Individuals on plant-based diets may also have lower calcium intake, particularly if dairy is excluded. Calcium can be sourced from fortified plant-based milks and yogurts, leafy greens, and tofu; however, bioavailability may be limited due to oxalates in certain plant foods [25]. Additionally, vitamin D deficiency poses a concern, particularly in regions with limited sunlight exposure, as few plant-based foods naturally contain vitamin D [25]. Fortified foods or supplements may be necessary to maintain bone health. This is prevalent in many dietary patterns, including omnivorous diets, as deficiencies and suboptimal levels are not limited to plant-based eating but are widespread across the general population. Notably, suboptimal vitamin D status is standard across dietary patterns [25]. While a plant-based diet can meet protein requirements, concerns regarding the adequacy of essential amino acids are largely outdated. Ensuring a variety of protein sources is key. However, in individuals with reduced kidney function and low protein intake, layering multiple therapeutic dietary modifications may increase the risk of insufficient intake of certain essential amino acids [38]. Nonetheless, consuming a variety of plant protein sources—such as legumes, nuts, seeds, and whole grains—across meals can ensure sufficient intake of all essential amino acids [38].

## 3. Impact of Plant-Based Diets on PKD

In the past, the role of plant-based diets was not very well-documented or studied. Historically understudied, plant-based diets have more recently been associated with potential improvements in CKD progression, including in PKD [20]. Evaluating the possible role of plant-based diets in mitigating cyst formation and progression is essential [39].

### 3.1. Impact on Primary Molecular Mechanisms in PKD

Although dysregulation of the mTOR pathway is studied and highlighted as a key contributor to cystogenesis, the primary molecular events following the loss of polycystin function remain incompletely understood. The initial trigger that links polycystin functional impairment to downstream pathways such as mTOR, cAMP, and altered cellular metabolism has yet to be fully elucidated [40]. As previously described, a well-established molecular mechanism involved in cystogenesis is the mTOR pathway [9,39,41].

Plant-based diets are generally associated with reduced protein intake, particularly from animal sources, which have been shown to stimulate the mTOR pathway. By lowering protein intake, particularly from animal meat, a plant-based diet can reduce hyperactivation of this pathway, potentially slowing cyst growth [22,42,43]. The research to date has primarily focused on meat consumption. In contrast, the evidence regarding dairy and other animal-based proteins is less consistent; these sources have not demonstrated the same association and, in some cohorts, have even been suggested to be protective [44]. However, PKD-specific data remain limited, and findings should therefore be interpreted with caution.

Another important pathway associated with PKD is impaired energy metabolism in kidney cells, which leads to cyst formation [45,46]. The AMP-activated protein kinase (AMPK) pathway is crucial for regulating cellular energy balance, and its dysfunction can worsen cyst growth [22,38,46,47]. Plant-based diets, which are often lower in fat and higher in fiber, may help improve insulin sensitivity and overall energy metabolism [12,20,22,42]. The AMPK pathway can be activated by nutrient signals, such as reduced caloric intake or plant-derived polyphenols, which promote cellular energy efficiency [12,36]. By improving energy metabolism, a plant-based diet can reduce cyst cell proliferation [12].

### 3.2. Impact on Secondary Pathways and Complications

Additionally, PKD is characterized by increased oxidative stress, which accelerates cyst formation and kidney damage [8,10]. Reactive oxygen species (ROS) generated in PKD cells cause oxidative damage to tissues, promoting inflammation and fibrosis [11,46]. A plant-based diet is rich in antioxidants (vitamins C, E, polyphenols, carotenoids, and flavonoids), which neutralize ROS and reduce oxidative damage [20,28,29]. Antioxidants from plant foods may decrease cellular damage, slow cyst growth, and preserve kidney function [48]. In addition to oxidative stress, inflammation is another component that drives cyst formation. The NF-κB (nuclear factor kappa-light-chain-enhancer of activated B cells) pathway plays a central role in promoting inflammation in PKD, releasing cytokines and growth factors that promote cyst expansion [9,41,45,46]. Anti-inflammatory compounds in plant foods (such as omega-3 fatty acids from seeds, polyphenols from fruits, and fibers) may inhibit the NF-κB pathway [42,49,50]. This reduces inflammatory cytokine production, limiting the immune response that accelerates cyst growth. Furthermore, as cysts grow in PKD, they disrupt normal kidney structure, leading to fibrosis and scarring. The transforming growth factor-beta (TGF-β) pathway is a major regulator of kidney fibrosis, promoting the deposition of extracellular matrix and scarring [51]. A plant-based diet may help reduce fibrosis through its anti-inflammatory and antioxidant effects. In addition, plant-based diets may modulate the TGF-β signaling pathway by providing compounds such as flavonoids, which inhibit fibrosis [52]. Less fibrosis is associated with a slower progression of kidney damage [51].

As CKD progresses, metabolic abnormalities worsen [39]. Elevated uric acid levels are common in PKD and can exacerbate kidney damage by promoting inflammation and oxidative stress, both of which stimulate cyst and stone formation [53]. Plant-based diets are naturally low in purines, which are precursors to uric acid [22,54]. A reduction in uric acid levels is associated with less inflammatory signaling and reduced cyst progression [22,53,54]. For patients receiving tolvaptan, a vasopressin V2 receptor antagonist that slows cyst growth, plant-based diets may offer complementary benefits by improving insulin sensitivity, reducing oxidative stress, and mitigating dietary acid load. Such dietary approaches could synergize with pharmacologic therapy to further slow disease progression, though dedicated clinical studies are needed to evaluate safety, efficacy, and potential interactions [55].

**Figure 1:**
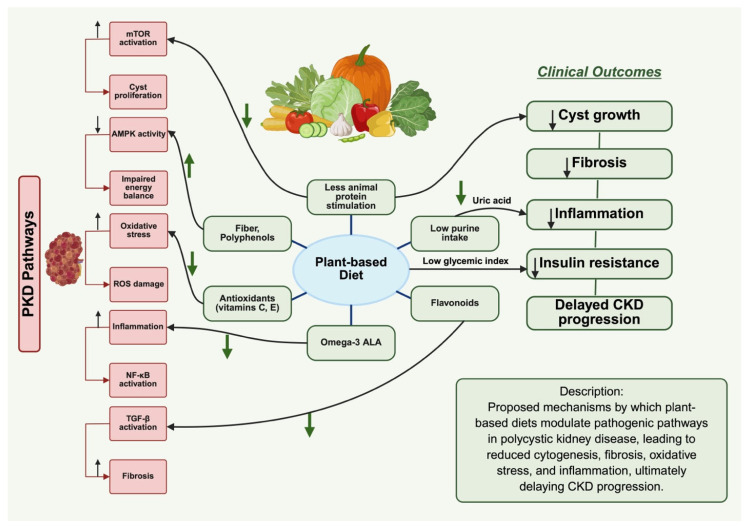
Illustrates the impact of plant-based diet on molecular mechanisms in polycystic kidney disease. Created in BioRender. Ebrahimi, N. (2025) https://BioRender.com/z58i164 (accessed on 8 October 2025). Red boxes represent pathogenic PKD pathways, including mTOR activation, oxidative stress, inflammation, and fibrosis. Green boxes denote dietary components and clinical outcomes associated with plant-based nutrition. Upward arrows (↑) indicate an increase or activation, while downward arrows (↓) indicate a decrease or inhibition of the corresponding pathway or process. Green arrows indicate the beneficial inhibitory effects of a plant-based diet on pathogenic mechanisms (e.g., ↓ decreased oxidative stress, ↓ decreased mTOR activation, ↓ decreased inflammation). Black arrows represent causal or sequential relationships that lead to downstream outcomes, such as reduced cyst growth, fibrosis, and delayed CKD progression.

Activation of the renin–angiotensin–aldosterone system (RAAS) in PKD contributes to cyst growth and kidney injury by promoting cell proliferation, fluid secretion, and fibrosis. RAAS inhibition has been shown to slow disease progression and reduce kidney damage in PKD models and patients [6]. The high potassium and low sodium content in plant-based diets help to regulate blood pressure [22,54]. Potassium counteracts the effects of sodium on blood pressure, and plant-derived nitrates (found in leafy greens) can dilate blood vessels [22,54]. This results in reduced RAAS activity, less kidney stress, and slower cyst growth. Additionally, insulin resistance is commonly observed in PKD patients and contributes to the progression of cyst formation through metabolic dysfunction [56,57]. The proposed molecular mechanism behind cyst progression is that increased insulin resistance stimulates the mTOR pathway, which is dysregulated in PKD [56,57]. Plant-based diets are rich in fiber and have a low glycemic index, which helps control blood sugar levels and improve insulin sensitivity [28]. Better glycemic control reduces the metabolic burden on the kidneys, slowing the pathological processes associated with cyst growth (Figure 1) [28,57].

Advanced glycation end products (AGEs), formed through the non-enzymatic glycation of proteins or lipids, significantly contribute to oxidative stress, inflammation, and tissue fibrosis, all of which are central to the PKD pathogenesis and progression of PKD. AGEs accumulate in kidney tissues and promote cellular injury through receptor-mediated pathways, exacerbating cyst expansion and fibrotic remodeling. Diet is a modifiable source of AGE exposure, with plant-based diets generally containing lower levels of AGEs compared to animal-based diets, particularly those subjected to high-temperature cooking methods. Therefore, a reduction in dietary AGE intake through plant-based nutrition may offer a therapeutic avenue to mitigate disease progression in PKD [58]. Furthermore, phosphorus metabolism in PKD warrants attention, especially in advanced stages where phosphate retention contributes to vascular calcification and CKD-mineral bone disorder. Phosphate derived from plant sources exists predominantly in the form of phytate, which is poorly absorbed in the human gastrointestinal tract due to a lack of endogenous phytase. In contrast, phosphorus from animal products and food additives is highly bioavailable, resulting in a greater phosphate burden. Thus, shifting toward plant-based sources of phosphorus may attenuate phosphate overload, offering additional renoprotective benefits in PKD management [59,60].

In addition to metabolic and inflammatory pathways, emerging evidence highlights the role of the gut–kidney axis and the intestinal microbiome in the pathogenesis and progression of PKD.

Trimethylamine N-oxide (TMAO), a gut microbiota-derived metabolite produced during the digestion of meat, has emerged as a key mediator of kidney and systemic inflammation. Elevated TMAO levels are associated with increased TNF-α expression, which promotes kidney inflammation, fibrosis, and the progression of CKD, while also contributing to cardiovascular risk and oxidative stress [61]. Plant-based diets are known to support a diverse gut microbiota and promote the production of beneficial metabolites such as short-chain fatty acids (SCFAs), including butyrate, which have anti-inflammatory and epithelial barrier-preserving properties. Additionally, plant-based nutrition is associated with a lower generation of protein-bound uremic toxins, such as indoxyl sulfate and p-cresyl sulfate, metabolites derived from the microbial fermentation of dietary protein. These toxins are predominantly cleared through active tubular secretion rather than glomerular filtration. Notably, in PKD, which is fundamentally a tubular disorder, recent data suggest that proximal tubular secretory function is disproportionately impaired relative to GFR, resulting in reduced clearance of these solutes even in early disease stages [62]. Therefore, dietary strategies that modulate the gut microbiome and limit the generation of uremic toxins may represent a novel therapeutic avenue for mitigating disease burden in PKD, particularly when coupled with approaches to support residual tubular secretion.

While the ketogenic diet has demonstrated short-term feasibility and promising outcomes in ADPKD, it is important to contextualize these findings within the broader landscape of dietary strategies under investigation. In contrast, plant-based diets emphasize a high intake of fruits, vegetables, whole grains, legumes, and healthy fats, and are typically lower in protein, especially animal-derived protein. Plant-based diets are rich in antioxidants, anti-inflammatory compounds, and fiber, and have been associated with improved insulin sensitivity, reduced oxidative stress, and favorable cardiovascular outcomes in CKD. Although both diets may modulate metabolic and signaling pathways relevant to cystogenesis, such as mTOR and AMPK, they do so via distinct nutritional profiles [39]. A growing area of interest is the development of plant-based ketogenic diets, which aim to induce ketosis using predominantly plant-derived fats and proteins, potentially offering metabolic benefits while mitigating some of the adverse effects observed with traditional animal-based ketogenic diets [39]. However, clinical data on such hybrid approaches remain sparse, and future studies are needed to evaluate their efficacy, safety, and sustainability in patients with ADPKD.

### 3.3. Clinical Evidence and Observational Studies

Emerging evidence suggests that cystic kidney cells in ADPKD exhibit altered cellular metabolism reminiscent of the Warburg effect, characterized by preferential glucose utilization via aerobic glycolysis rather than oxidative phosphorylation. This metabolic shift supports increased proliferation and cyst growth, highlighting the potential of dietary strategies, including plant-based or carbohydrate-modulated diets, to influence cellular energy metabolism and disease progression [63]. Observational studies have explored ketogenic diets in ADPKD, reporting reductions in kidney volume growth and improvements in metabolic parameters in the short term. While promising, these findings are limited by small sample sizes, short follow-up, and potential risks associated with high protein or animal-fat content, particularly in patients with reduced kidney function [64,65]. The MDRD trial demonstrated that moderate protein restriction slowed the progression of CKD in adults, providing foundational evidence for protein-modulated dietary interventions. Although ADPKD-specific data are limited, these findings support the rationale for controlled protein intake, particularly from plant-based sources, to mitigate cyst growth while maintaining nutritional adequacy [66]. Clinical evidence also supports the potential benefits of plant-based diets in ADPKD. A recent study investigated the association between adherence to a plant-based diet and kidney function among 106 patients with ADPKD. Dietary intake was assessed using the overall plant-based diet index (PDI), healthful PDI (hPDI), and unhealthful PDI (uPDI). Patients with advanced CKD (eGFR < 60 mL/min/1.73 m^2^) had lower overall PDI and hPDI scores and higher uPDI scores than those with early CKD. Importantly, higher adherence to an hPDI was inversely associated with advanced CKD (OR: 0.117; 95% CI: 0.039–0.351; *p* < 0.001), while adherence to a uPDI was positively associated with advanced CKD (OR: 8.450; 95% CI: 2.810–25.409; *p* < 0.001). The hPDI was also negatively correlated with markers of systemic inflammation, including the neutrophil-to-lymphocyte and platelet-to-lymphocyte ratios. These findings indicate that greater adherence to a healthful plant-based diet may be associated with improved kidney function and reduced inflammation in ADPKD patients, supporting the potential role of dietary strategies in disease management [55].

Longitudinal cohort studies, such as the CRIC and NHS cohorts, have shown that higher adherence to plant-based or Mediterranean diets is associated with slower kidney function decline and lower all-cause mortality in CKD patients. Though PKD-specific trials are limited, the biological mechanisms shared between PKD, and general CKD suggest that these findings may be translatable to the PKD population [24].

Preclinical studies in animal models highlight several dietary and molecular mechanisms relevant to PKD progression, as summarized in Table 1.

## 4. Conclusions

PKD is a complex genetic disorder with significant implications for kidney function and overall health. The progressive formation of cysts, driven by molecular mechanisms such as the mTOR and AMPK pathways, along with oxidative stress and inflammation, underlines the need for holistic management strategies. Plant-based diets, rich in antioxidants, fiber, and anti-inflammatory compounds, offer a promising avenue for mitigating some of the adverse outcomes associated with PKD. By modulating pathways associated with cyst growth and reducing oxidative damage, a plant-based diet may slow disease progression and preserve kidney function. Moreover, the potential cardiovascular and metabolic benefits of such diets, including improved blood pressure regulation and enhanced insulin sensitivity, further emphasize their relevance for patients with PKD. However, dietary interventions should be personalized to address potential nutritional deficiencies, such as vitamin B12, iron, and omega-3 fatty acids, which are common concerns in plant-based diets. Supplementation and careful planning are crucial to ensure that these diets are nutritionally adequate and kidney friendly. Emerging evidence suggests that plant-based diets, combined with existing medical therapies and healthy lifestyle habits such as limiting alcohol intake, could play a pivotal role in improving the quality of life and clinical outcomes for patients with PKD. Further PKD-specific clinical trials, particularly those comparing plant-based dietary interventions to standard care, are warranted to establish efficacy, optimize nutritional strategies, and inform the integration of dietary therapy into comprehensive management plans for this population.

### Limitations

This review synthesizes emerging mechanistic and observational evidence regarding the potential benefits of plant-based diets in PKD. It does not include original data, nor does it represent a systematic review. The proposed effects on cystogenesis, metabolic pathways, and clinical outcomes remain largely theoretical and supported predominantly by preclinical or non-PKD-specific studies. Consequently, this review should be interpreted as an expert opinion that highlights biologically plausible concepts rather than evidence-based dietary recommendations. Well-designed interventional trials in patients with ADPKD are needed to determine whether these hypotheses translate into meaningful clinical benefit. Moreover, recent findings emphasize that the ultra-processed food category is highly heterogeneous, and certain plant-based ultra-processed products, such as plant-based milks, meat analogs, and margarine, may confer cardiometabolic advantages compared to their unprocessed animal-based counterparts. Therefore, generalizations regarding the health impact of ultra-processed foods should be made with caution, and future research should account for this heterogeneity when evaluating plant-based dietary interventions in PKD [67].

## Figures and Tables

**Table 1:** Preclinical evidence on dietary and molecular pathways in PKD. ↑ indicates an increase or upregulation; ↓ indicates a decrease or downregulation in the corresponding parameter or pathway.

Model/Study	Intervention	Findings	Mechanism/Pathway	Reference
ARPKD animal models	Genetic PKHD1 mutations	Elevated cAMP promotes cystogenesis	V2R hyperactivity → ↑cAMP → activation of MAPK and mTOR pathways	Cordido et al. [41] 2021; Wang et al. [45] 2005
PCK rat model	V2 receptor antagonists (OPC-31260, OPC-41061)	Reduced cyst development and preserved kidney architecture	Blockade of vasopressin-V2R signaling → ↓cAMP	Wang et al. [45] 2005
Mouse PKD model	Induced microcrystal deposition	Tubule dilation accelerated cyst growth	Crystal deposition promotes tubular injury and cystogenesis	Torres et al. [18] 2019
Rat PKD model	Soy protein diet vs. animal protein	Attenuated cyst growth	Modulation of growth signaling; anti-inflammatory/antioxidant effects	Ogborn et al. [50] 1998
Transgenic mouse model	TGF-β1 overexpression	Increased kidney fibrosis and accelerated functional decline	TGF-β–mediated extracellular matrix deposition and scarring	Zhang et al. [51] 2020
Fibrosis models	Plant flavonoids	Reduced fibrosis	Inhibition of TGF-β signaling; anti-inflammatory properties	Wang [52] 2023
Animal CKD models (gut–kidney axis)	Meat-based vs. plant-based diets	Meat diet: ↑TMAO, uremic toxins, inflammation; Plant diet: ↑SCFAs, improved gut barrier	Modulation of microbiota-derived metabolites; ↓TNF-α, ↓fibrosis	Zixin et al. [61] 2022

## Data Availability

No new data were created or analyzed in this study. Data sharing is not applicable to this article.
